# Hyperspectral and Chlorophyll Fluorescence Analyses of Comparative Leaf Surfaces Reveal Cellular Influences on Leaf Optical Properties in Tradescantia Plants

**DOI:** 10.3390/cells13110952

**Published:** 2024-05-30

**Authors:** Renan Falcioni, Werner Camargos Antunes, Roney Berti de Oliveira, Marcelo Luiz Chicati, José Alexandre M. Demattê, Marcos Rafael Nanni

**Affiliations:** 1 Department of Agronomy, State University of Maringá, Av. Colombo, 5790, Maringá 87020-900, Paraná, Brazil; wcantunes@uem.br (W.C.A.); rboliveira@uem.br (R.B.d.O.); mlchicati@uem.br (M.L.C.); mrnanni@uem.br (M.R.N.); 2 Department of Soil Science, Luiz de Queiroz College of Agriculture, University of São Paulo, Av. Pádua Dias, 11, Piracicaba 13418-260, São Paulo, Brazil; jamdemat@usp.br

**Keywords:** anthocyanins, biochemical and biophysical, hyperspectral and microscopic, hyperspectral vegetation index, principal component analysis, VIS-NIR-SWIR

## Abstract

The differential effects of cellular and ultrastructural characteristics on the optical properties of adaxial and abaxial leaf surfaces in the genus *Tradescantia* highlight the intricate relationships between cellular arrangement and pigment distribution in the plant cells. We examined hyperspectral and chlorophyll *a* fluorescence (ChlF) kinetics using spectroradiometers and optical and electron microscopy techniques. The leaves were analysed for their spectral properties and cellular makeup. The biochemical compounds were measured and correlated with the biophysical and ultrastructural features. The main findings showed that the top and bottom leaf surfaces had different amounts and patterns of pigments, especially anthocyanins, flavonoids, total phenolics, chlorophyll-carotenoids, and cell and organelle structures, as revealed by the hyperspectral vegetation index (HVI). These differences were further elucidated by the correlation coefficients, which influence the optical signatures of the leaves. Additionally, ChlF kinetics varied between leaf surfaces, correlating with VIS-NIR-SWIR bands through distinct cellular structures and pigment concentrations in the hypodermis cells. We confirmed that the unique optical properties of each leaf surface arise not only from pigmentation but also from complex cellular arrangements and structural adaptations. Some of the factors that affect how leaves reflect light are the arrangement of chloroplasts, thylakoid membranes, vacuoles, and the relative size of the cells themselves. These findings improve our knowledge of the biophysical and biochemical reasons for leaf optical diversity, and indicate possible implications for photosynthetic efficiency and stress adaptation under different environmental conditions in the mesophyll cells of Tradescantia plants.

## 1. Introduction

Leaves, the primary photosynthetic organs of higher plants, exhibit complex interactions between their structural composition and optical properties which are essential for photosynthesis [1,2,3]. Notably, pigments, such as anthocyanins and phenolic compounds, play critical roles in determining foliar optical properties, modifying light absorption, enhancing reflectance, and adjusting transmittance of energy in leaves [4,5,6]. These pigments not only contribute to the unique optical signatures analysed via spectral data, but also enhance photoprotection and light management, which are crucial for photosynthesis [2,7]. In *Tradescantia* species, pronounced pigmentation, particularly that of anthocyanins, aids in light attenuation and stress mitigation under intense light conditions [8,9].

*Tradescantia spathacea*, commonly known as Boat Lily, is an evergreen herbaceous perennial belonging to the Commelinaceae family, indigenous to southern Mexico and Guatemala. This plant is characterised by its clumping growth habit and broad, linear leaves that display a remarkable chromatic shift from green on the upper (adaxial) surface to purple on the lower (abaxial) surface. In contrast, *Tradescantia pallida*, commonly referred to as a purple heart, is notable for its deep, uniform purple pigmentation that saturates the entirety of its foliage, although it typically shows a gradient from a darker tone on the adaxial side to a slightly lighter purple on the abaxial side [8,9,10,11].

Plant leaves have contrasting functions related to how they are organised at the cellular level, affecting their interactions with light and fluorescence properties. This is especially noticeable in species such as *Ophiopogon planiscapus*, *Euphorbia pulcherrima*, *Codiaeum variegatum*, *Passiflora edulis*, *Tradescantia spathacea*, *Tradescantia pallida* and others, where the location and chemical nature of cellular pigments, along with the structural configuration of leaf tissues, are the principal factors. The epidermis is the outermost protective layer, which protects plants from environmental stressors, controls light entry, optimises photosynthetic efficiency, and prevents photodamage to underlying tissues [12,13]. Under the epidermis, the hypodermis provides additional structural support and helps scatter light, thus improving the optical properties of the leaf, which influence light absorption and reflection. The chlorenchyma, composed of chlorophyll-bearing cells, is vital for photosynthesis [12]. Its structural arrangement strongly affects how well light is captured and converted into chemical energy, and influences chlorophyll *a* fluorescence (ChlF) dynamics [11,14]. Moreover, chloroplasts within the chlorenchyma cells, which have light-harvesting complexes and a photosynthetic electron transport chain, are essential for photosynthesis in deeper layers [2,15,16]. The location and density of these chloroplasts are key factors in the light absorption efficiency and functional performance of photosystem II, as shown by ChlF kinetics [17,18]. Studying these cellular structures helps understand the complex relationship between cellular architecture and plant physiological responses, enhancing our knowledge of how plants manage light and perform photosynthesis under different shade or sunlight environmental conditions [2,19,20].

The morphology, structure, and ultrastructure of leaf cells, including the number and arrangement of chloroplasts, significantly impact their ability to capture and use light, as indicated by the VIS-NIR-SWIR spectral curves [21,22,23,24]. These spectral techniques are essential for investigating how visible (VIS), near-infrared (NIR), and shortwave infrared (SWIR) light interacts with cellular structures, revealing intricate details about plant physiological responses to electromagnetic radiation [22,23,25]. Variations in chloroplast structures, such as the number of granum stacks and thylakoid arrangements, are important for maximising the light absorption and efficiency of the electron transport chain. Advanced microscopic techniques, such as electron microscopy, are key to visualising these subtle structural changes and provide a deeper understanding of how these structures function at the microscopic level [26,27]. These microscopic structures are overly sensitive to biophysical and biochemical stimuli, which reflects the plasticity of plants in adjusting to environmental changes through genetic variation and phenotypic flexibility. For example, VIS-NIR-SWIR spectroscopy and detailed microscopic analysis revealed complex interactions between plant structural adaptations and their environmental interactions, improving our knowledge of adaptability efficiency in plants [2,24,25,26].

Overall, the integration of correlation by hyperspectral, chlorophyll *a* fluorescence and microscopic techniques demonstrated that pigment distribution and leaf architecture synergistically affected the light management strategies of *Tradescantia* species [8,10,11]. By examining spectral indices and cellular details, different mechanisms by which structural modifications at the cellular level enhance light-energy management in plants can be identified, for example, deconvolution or hyperspectral vegetation index [24,28]. Specifically, chlorophyll fluorescence kinetics provide insights into the dynamic changes in the light-absorbing and energy-dissipating functions of chlorophyll *a* under variable light conditions.

In this study, VIS-NIR-SWIR hyperspectral and chlorophyll *a* fluorescence techniques were employed to acquire precise spectral profiles that correlate cellular and ultrastructural data with the physiological status of plants. This method facilitates an in-depth analysis of the influence of cellular composition and arrangement on the optical properties of leaves, and improves understanding of the modulation of light across both standard and nonstandard leaves, as well as analysing the spectral and fluorescence curves on both adaxial and abaxial surfaces [9,11,14,29]. By integrating detailed microscopic analyses of cellular structures with advanced spectral techniques, we aim to enhance our understanding of the complex interplay between plant cell morphology and function [11,28,30].

**Figure 1:**
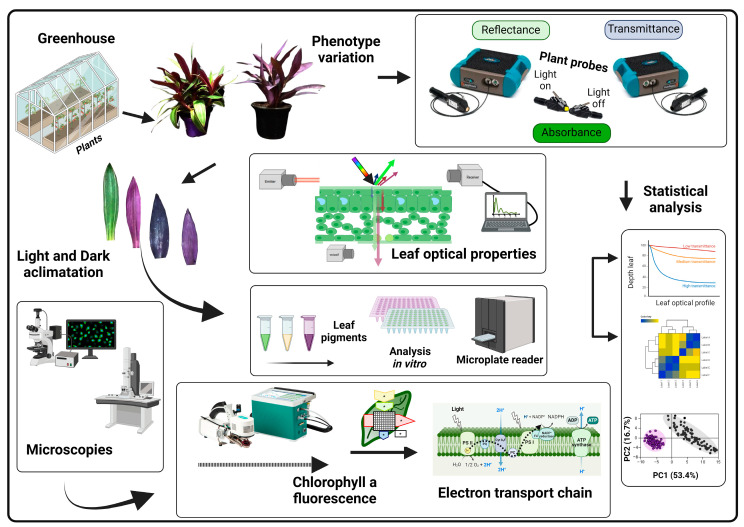
Flowchart illustrating the analysis of leaf optical properties, changes in pigment composition, leaf structure, and ultrastructure, along with chlorophyll *a* fluorescence and statistical analyses in *Tradescantia spathacea* (L.) Olof Swartz and *Tradescantia pallida* (Rose) D.R. Hunt., with variegated leaves. Plants were cultivated in a greenhouse, and their leaves were assessed using a novel method with two hyperspectral sensors to acquire reflectance, transmittance, and absorbance spectra. Leaves were analysed to evaluate changes in their optical properties [2,19]. The biochemical components of the leaves were measured in polar and apolar phases [7]. In addition, modifications in leaf structure and ultrastructure were examined using light microscopy as well as scanning and transmission electron microscopy techniques. Leaves were adapted to both light and dark conditions, and chlorophyll *a* fluorescence factors were measured using the JIP test dataset, focusing on observing changes in the electron transport chain. Finally, the data were subjected to univariate and multivariate analyses. The flow of analysis was considered starting from the growth of the plants and transitioning from non-destructive analyses to destructive analyses. Created with BioRender.com.

Based on the observed differences in photosynthetic function and structure between leaf tissues, we hypothesised that variations in chlorophyll fluorescence (ChlF) and optical properties between the adaxial and abaxial sides of bifacial leaves are primarily due to structural differences. We anticipate that these structural variations will also influence the shape of the hyperspectral vegetation index (HVI) data used to align the optical properties of leaves [2,31]. To evaluate this hypothesis, we analysed the structure, spectroscopy, and ChlF curves of the adaxial and abaxial sides of leaves *in vivo*. Our goal was to elucidate the differences in the HVI curves and assess the potential effects of leaf structure and ultrastructure on *Tradescantia* species, using both optical and electron microscopy (Figure 1).

## 2. Materials and Methods

### 2.1. Plant Materials and Experimental Design

*Tradescantia spathacea* (L.) Olof Swartz and *Tradescantia pallida* (Rose) D.R. Hunt, a horticultural plant with green and purple leaves, was grown in 2-L pots with MecPlant^®^ commercial substrate (MecPrec Ind., Telêmaco Borba, Paraná, Brazil) and NPK fertiliser (10-10-10; 1 g per pot). The plants were kept in a greenhouse under natural light conditions at a temperature of 21–26 °C and a humidity level of 50–70% [26]. A 14-h light cycle was maintained, with the light intensity changing throughout the day. The plants were watered twice daily at 8 a.m. and 6 p.m. to standardise the water conditions. Young, fully opened leaves were chosen for various analyses, with 200 leaf samples collected to evaluate their hyperspectral reflectance, transmittance, absorbance, and chlorophyll *a* fluorescence for leaf optical properties, biochemical profiles, leaf cell structure, and ultrastructure [32]. The samples were collected immediately before the analyses to ensure consistency in data collection. All measurements were conducted between 11 a.m. and 1 p.m., unless otherwise stated in the experimental modifications. Other procedures, such as material fixation, were carried out after the collection of hyperspectral data. Fluorescence and pigments were extracted and quantified sequentially, as described below (Figure 1).

### 2.2. Spectral Characterisation of Leaf Optical Properties through Hyperspectral Analysis

The interaction of cells in leaves with reflectance (R) and transmittance (T) properties *in vivo* was measured quantitatively using a novel method developed by Falcioni et al. (2023) [2] with a FieldSpec^®^ 3 spectroradiometer from Analytical Spectral Devices (ASD), Inc., Longmont, CO, USA [31,32,33]. This instrument, coupled with an ASD Contact PlantProbe^®^ (ASD Inc., Longmont, CO, USA) with 10 mm diameter, features a sophisticated detector array consisting of 512 InGaAs photodiodes, enabling highly accurate spectral data collection from 350 to 2500 nm. To reduce atmospheric effects during data collection, an ASD PlantProbe^®^ leaf clip calibrated with standard White Spectralon^®^ reference plates (Labsphere Inc., Longmont, CO, USA) was used. The upper surface of the leaf was exposed to a powerful light source integrated into the plant probe, whereas the lower surface was assessed using another probe, with its light source turned off. Simultaneous reflectance (R) and transmittance (T) measurements were performed at different wavelengths, with each leaf sample undergoing an average of 50 repeated measurements to obtain a consistent spectral profile. Absorbance (A) was calculated using the formula A = 1 − (R + T). The sensor settings were adjusted to 1000 and 1800 nm using the ViewSpec Pro software version 6.2 (ASD Inc., Boulder, CO, USA). In addition, this study also included data on spectral curves and leaf pigments assessed under *in vitro* conditions, covering the wavelength range of 350–750 nm [31,32,33].

### 2.3. Assessment of Leaf Tissue Composition

#### 2.3.1. Measurement of Chlorophyll and Carotenoids

We used a modified version of the method by Gitelson and Solovchenko (2018) [34] to measure the concentrations of chlorophyll *a*, chlorophyll *b*, total chlorophyll (*a* + *b*), and carotenoids, including carotenes and xanthophylls. We measured the absorbance of the methanol extract from the apolar phase of the chloroform/methanol (2:1) extraction at wavelengths of 470, 652, and 665 nm [35]. The concentrations of these pigments were calculated using the formulas and final concentrations were expressed in mg cm^−2^ and mg g^−1^ [35].

#### 2.3.2. Measurement of Anthocyanins and Flavonoids

To measure anthocyanins, we acidified the water-methanol phase with hydrochloric acid and measured its absorbance at λ530 nm using the molar absorption coefficient of Gitelson & Solovchenko (2018) [34]. To measure flavonoids, we examined the polar fraction of the methanol extract at a wavelength of 358 nm. The molar absorption coefficients were applied according to the method described by Gitelson and Solovchenko (2018) [34].

#### 2.3.3. Evaluation of Antioxidant Capacity

To evaluate the total antioxidant capacity of the samples, a DPPH assay adapted from the method of Llorach, Martínez-Sánchez, Tomás-Barberán, Gil, and Ferreres (2008) [36] was used. For this assay, we mixed the methanolic extract with a 1 mM solution of DPPH and measured the absorbance of the mixture after incubation in a microplate reader.

#### 2.3.4. Measurement of Phenolic Compounds in Leaves

The soluble phenolic compounds were measured using a revised version of the method that Ragaee (2006) [37] reported. The assay involved mixing the methanolic extract with Folin-Ciocalteu reagent, sodium carbonate (Na_2_CO_3_), and deionised water. We then incubated the mixture in the dark before centrifuging to separate the phases. The absorbance of the supernatant that was obtained was determined at 725 nm wavelength.

### 2.4. OJIP Chlorophyll a Fluorescence Transient

Leaf measurements were performed using an infrared gas exchange analyser (IRGA; LI-6800, LI-COR Inc., Lincoln, NE, USA) equipped with a multiphase flash fluorometer (LI-6800-01; LI-COR Inc.) [2,19]. The IRGA was configured for fluorescence measurements. The following specific conditions for analysis were used in setup: CO_2_ concentration of 400 µmol mol^−1^, 60% relative humidity in the sample chamber, a flow rate of 700 µmol s^−1^, a fan speed of 10,000 rpm, and sample heat exchanger temperature of 25 °C within a 6 cm^2^ chamber. The sensor was operated above 720 nm, specifically to detect chlorophyll fluorescence to photosystem II (PSII).

Each leaf was subjected to a 12-h dark acclimation period prior to the measurements. During the experiments, leaves were secured in a clipping chamber where a saturating light pulse of 15,000 µmol m^−2^ s^−1^ was applied for 1 s in the induction mode, ensuring the closure of all reaction centres. The methods and formulas for calculating the JIP test parameters were taken from the literature. The JIP test parameters related to the electron transport chain in plants were measured using the Biolyzer software (version 4.0; Laboratory of Bioenergetics, University of Geneva, Geneva, Switzerland) [38,39,40].

### 2.5. Preparation and Microscopic Analyses

#### 2.5.1. Sample Preparation for Microscopy

The leaves were meticulously prepared for examination by optical microscopy (OM), scanning electron microscopy (SEM), and transmission electron microscopy (TEM) [27]. The samples were cut into cubic millimetres using a scalpel blade on a paraffin-coated Petri dish and immediately immersed in a droplet of fixative solution to prevent damage and ensure rapid preservation. The fixative solution used was a modified Karnovsky solution, which was originally described by Karnovsky (1965) [41], comprising 2.5% glutaraldehyde and 2% paraformaldehyde in 0.05 M cacodylate buffer at pH 7.2. After six hours in this solution, the samples were fixed with a mixture of 1% osmium tetroxide and 1.6% potassium ferrocyanide in the same buffer for better cell and organelle contrasts [27].

After fixation, the samples were contrasted for 12 h with 0.5% uranyl acetate, followed by a graded dehydration series using acetone at concentrations ranging from 30 to 100%, with three cycles at the highest concentration. Portions of the samples were reserved for SEM-specific processing. The remaining samples were infiltrated and embedded in Spurr low-viscosity epoxy resin. The resin blocks were sectioned into semi-thin (1 μm) and ultrathin (70 nm) sections using an MTX Powertome X ultramicrotome (Boeckeler Instruments, RCM Products, Egham, UK). Sectioning was performed using both glass and diamond knives to meet the different thickness specifications. All reagents were of electron microscopy grade and obtained from Sigma-Aldrich (Sigma-Aldrich Inc., St. Louis, MO, USA) or Electron Microscopy Sciences (EMS, Hatfield, PA, USA).

#### 2.5.2. Optical Microscopy

For optical microscopy (OM), leaf sections with a thickness of 1 μm were stained with 1% toluidine blue solution in borax buffer [42]. To improve the staining process, the samples were briefly heated on a hot plate at 70 °C for 5 s. Observations were performed using a Leica ICC50 optical microscope (Leica Microsystems, Wetzlar, Germany). Various anatomical features, including the characteristics of the adaxial and abaxial surfaces, stomatal distributions, thicknesses of the adaxial and abaxial epidermal layers, and mesophyll structures, were quantitatively assessed. Dimensional analysis was performed using ImageJ version 1.54g software (National Institutes of Health, New York, NY, USA), while contrast enhancement in false colours and further quantitative assessments were conducted using the Image-Pro-Plus^®^ version 4.5 software (Media Cybernetics Inc., Rockville, MD, USA).

#### 2.5.3. Scanning Electron Microscopy

Leaf samples were prepared using the critical point drying (CPD) method with a CPD-030 device (Bal-Tec AG, Balzers, Liechtenstein). After drying, the samples were mounted on aluminium stubs using double-sided carbon adhesive tape and sputter-coated with gold, and a current of 50 mA was applied for 150 s using an MED010 Balzer evaporator (Bal-Tec AG Balzers, Liechtenstein, DEU). Observations were conducted using a Quanta 250 scanning electron microscope (Thermo Fisher Scientific, Hillsboro, OR, USA) operated at either 15 kV or 20 kV. Scanning and imaging were performed using the microscope’s integrated FEI version 5.2.6 software (Thermo Fisher Scientific, Hillsboro, OR, USA). This approach enabled detailed assessments of stomatal density on both the adaxial and abaxial surfaces of the leaves, as well as comprehensive characterisation of leaf trichomes. Image-Pro-Plus^®^ version 4.5 software (Media Cybernetics Inc., Rockville, MD, USA) was used to perform both quantitative and qualitative analyses of the images obtained.

#### 2.5.4. Transmission Electron Microscopy

Ultrathin sections, either 60 nm or 70 nm thick, were prepared and placed onto copper mesh grids rated at 300 mesh. The sections underwent contrast enhancement using 3% uranyl acetate for 30 min, followed by a 15-min treatment with lead citrate, following the method developed by Reynolds (1963) [43]. Observations were made using a JEOL JEM-1400 (JEOL Inc., Peabody, MA, USA) transmission electron microscope operating at 80 kV, which was equipped with a digital imaging system (Leica Microsystems, Inc., Deerfield, IL, USA). This configuration allowed for a detailed examination of cellular ultrastructures, including chloroplasts, thylakoid membranes, mitochondria, vacuoles, cytoplasmic components [20]. Both quantitative and qualitative analyses were performed using the Image-Pro Plus^®^ version 4.5 software (Media Cybernetics Inc., Rockville, MD, USA).

### 2.6. Hyperspectral Vegetation Indices Using Optimal Wavelengths for Biophysical Cell Parameters

To refine the accuracy of assessments based on chlorophyll *a* fluorescence and absorbance data, essential hyperspectral bands were identified through the application of the normalised difference vegetation index formula, as delineated in Equation (1) by Crusiol et al. (2023) [24]. This methodology facilitated the derivation of distinct hyperspectral vegetation indices (HVIs) relative to the biophysical indices of plant cells. Subsequent analyses involved correlating each HVI with cross-sectional data pertinent to the anatomical, structural, and ultrastructural features of plant cells. The Pearson correlation coefficient and the coefficient of determination (R^2^) were used to establish quantitative correlations. These were analysed using custom-made IDL programming and spectral data acquisition, covering a range of 350 to 2500 nm. Analytical results were visualised using Surfer^®^ version 16.6software (Golden Software, LLC., Golden, CO, USA) to generate a detailed contour map of the data.

(1) $$HVI=\frac{Wavelength\;1-Wavelength\;2}{Wavelength\;1+Wavelength\;2}$$

### 2.7. Univariate and Multivariate Analyses

Variability of variances across all variables was assessed using Bartlett’s test, which showed that there was no need for data transformation. Quantitative outcomes were analysed using paired t tests, and the results are presented as the mean ± standard error (SE). The significance threshold was set at *p* < 0.01 to determine statistical significance. Pearson’s correlation coefficient was used to examine the relationships among variables when appropriate. Statistica^®^ 10.0 (StatSoft, Inc., Tulsa, OK, USA), SigmaPlot^®^ 10.0 (Systat Software, Inc., San Jose, CA, USA), and the R statistical package (R Core Team, 2020) were used for all univariate and multivariate statistical analyses.

The dataset related to growth parameters was subjected to principal component analysis (PCA) using Unscrambler X software, version 10.4 (CAMO Software, Oslo, Norway). To ensure analytical validity, a significance level of *p* < 0.01 was applied. The best number of principal components was chosen based on the first maximum of the cumulative explained variance following Zar’s (2010) [44]. PCA also enabled the grouping of two species, *T. spathacea* and *T. pallida*, along with the identification of vectors for each group corresponding to each species component (Jolliffe and Cadima, 2016) [45]. This analytical method provides detailed information on the interdependencies of the hyperspectral and fluorescence kinetic curves within the studied species.

## 3. Results

### 3.1. Morphological Characteristics

**Figure 2:**
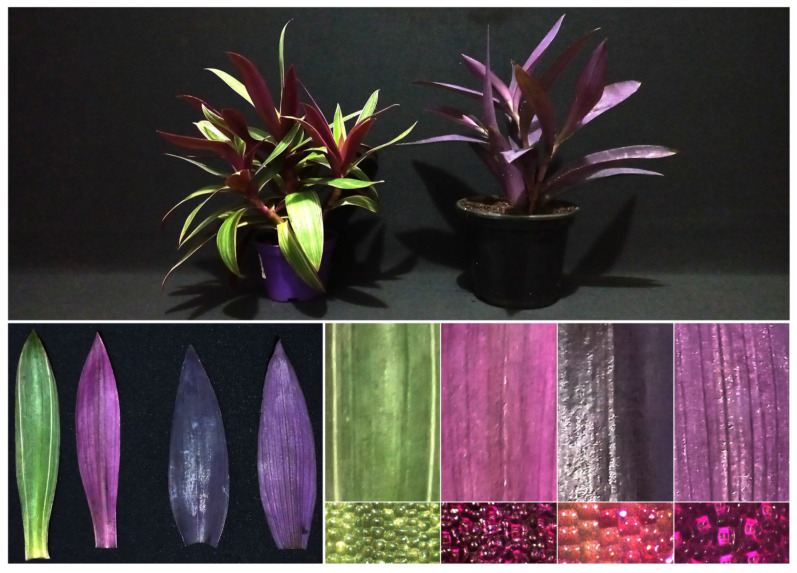
Images of *Tradescantia spathacea* (L.) Olof Swartz and *Tradescantia pallida* (Rose) D.R. Hunt plants. The variegated leaves evaluated in this study displayed significant variations in the bioaccumulation of pigments (i.e., the accumulation of pigments based on secondary metabolism). Upper panel: Details of the lateral profile of plants. Lower panel: details of the plant leaves, including leaf surface and paved epidermal cells.

Figure 2 shows representative specimens of *Tradescantia spathacea* (L.) Olof Swartz and *Tradescantia pallida* (Rose) D.R. Hunt. These species exhibit distinctive green and purple hues, with morphological differences between their adaxial and abaxial leaves (Figure 2).

*T. spathacea* displays a unique colour transition from green on the adaxial leaf surface to purple on the abaxial leaf surface. Conversely, *T. pallida* maintained a consistent purple hue on both leaf surfaces, with a more intense shade on the adaxial surface (Figure 2, top and bottom panels).

Stereoscopic microscopic analysis revealed variations in epidermal cell patterns between species. In *T. spathacea*, epidermal cells are irregular in shape and size, and pigments are unevenly distributed. In contrast, *T. pallida* exhibits epidermal cells that are more uniform in shape and arrangement with a more distinct and consistent pigment distribution. These observations highlight significant differences in cell morphology and pigment processing, which contribute to the distinct visual appearance of each species (Figure 2).

### 3.2. Microscopic Epidermal Architecture of Tradescantia Species

**Figure 3:**
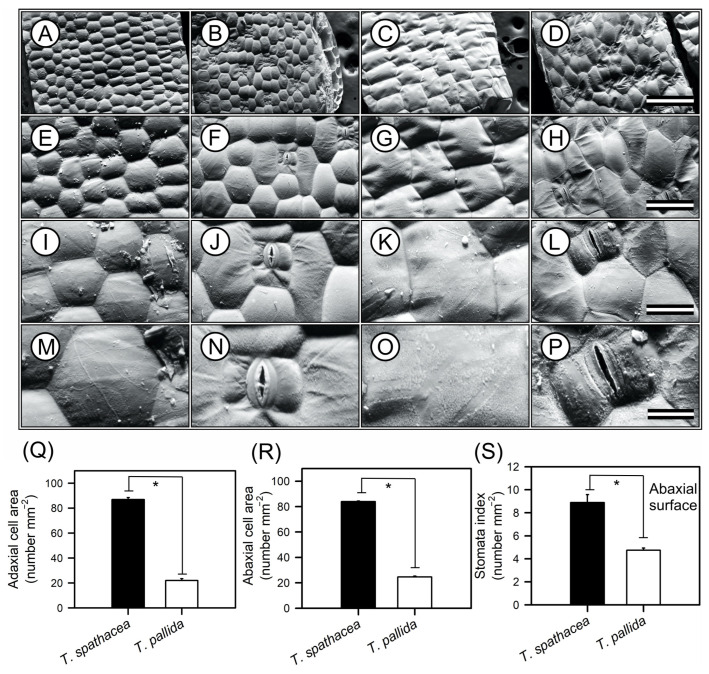
Representative scanning electron microscopy (SEM) images of the adaxial and abaxial surfaces of *Tradescantia spathacea* (L.) Olof Swartz and *Tradescantia pallida* (Rose) D.R. Hunt plants. (**A**,**E**,**I**,**M**) Adaxial surface of *T. spathacea*. (**B**,**F**,**J**,**N**) Abaxial surfaces of *T. spathacea*. (**C**,**G**,**K**,**O**) Adaxial surfaces of *T. pallida* species. (**D**,**H**,**L**,**P**) Abaxial surface of *T. pallida*. (**Q**) Adaxial cell surface area. (**R**) Abaxial cell surface area. (**S**) Stomatal indices of the abaxial surfaces of both the species. Scale bars = 500 μm, 200 μm, 100 μm, and 50 μm, from top to bottom. Bars with asterisks show significant differences by Student’s *t* test (*p* < 0.01). Mean ± SE. (*n* = 100).

Scanning electron microscopy (SEM) images of the epidermal structures of *T. spathacea* and *T. pallida* are shown in Figure 3. High-resolution SEM images revealed differences in the morphology of both adaxial and abaxial leaf surfaces (Figure 3A–P). The *T. spathacea* adaxial epidermis exhibited a cellular area density of approximately 86.85 cells mm^−2^ (Figure 3A,E,I,M,Q), while the abaxial surface showed a slightly reduced density of 83.96 cells mm^−2^ (Figure 3B,F,J,N,R). In contrast, *T. pallida* possessed a lower density of 21.99 cells mm^−2^ adaxially (Figure 3C,G,K,O,Q) and 24.63 cells mm^−2^ (Figure 3D,H,L,P,R). SEM revealed a more compact epidermal structure in *T. spathacea* than in *T. pallida* (Figure 3). For example, both species exhibited hexagonal-shaped cells with concavity facing the inner side of the mesophyll, forming convex lenses towards the external environment and concave towards the interior of the leaf mesophyll (Figure 3).

Stomatal indices on the lower surfaces could also distinguish between species (see Figure 3N,P). For example, *T. spathacea* had a greater index (8.89) than T. *pallida* (4.75), according to statistical analyses (Figure 3S). Both species lack stomata on their upper surfaces, which indicate that they are hypostomatic. Statistical analyses, performed with a confidence interval of 99% (*p* < 0.01), confirmed the significance of differences between species (Figure 3).

From a structural perspective, the cells on the upper and lower surfaces of *T. spathacea* had a hexagonal shape, whereas those on *T. pallida* exhibit a rectangular shape. These cells are tightly packed and arranged at almost 90° angles on the upper surface but are more loosely packed and still have clear outlines on the lower surface of both species (Figure 3A–P).

However, the features of the lower surfaces include paracytic stomata, which have guard cells that are usually longer than the adjacent cells and parallel to the abaxial surface (Figure 3J,L). The subsidiary cells, as shown, are also parallel to the guard cells and assist with the functioning and maintenance of the stomata but do not directly participate in the opening and closing of the stomata (Figure 3J,N,L,P).

### 3.3. Leaf Anatomy Structures

**Figure 4:**
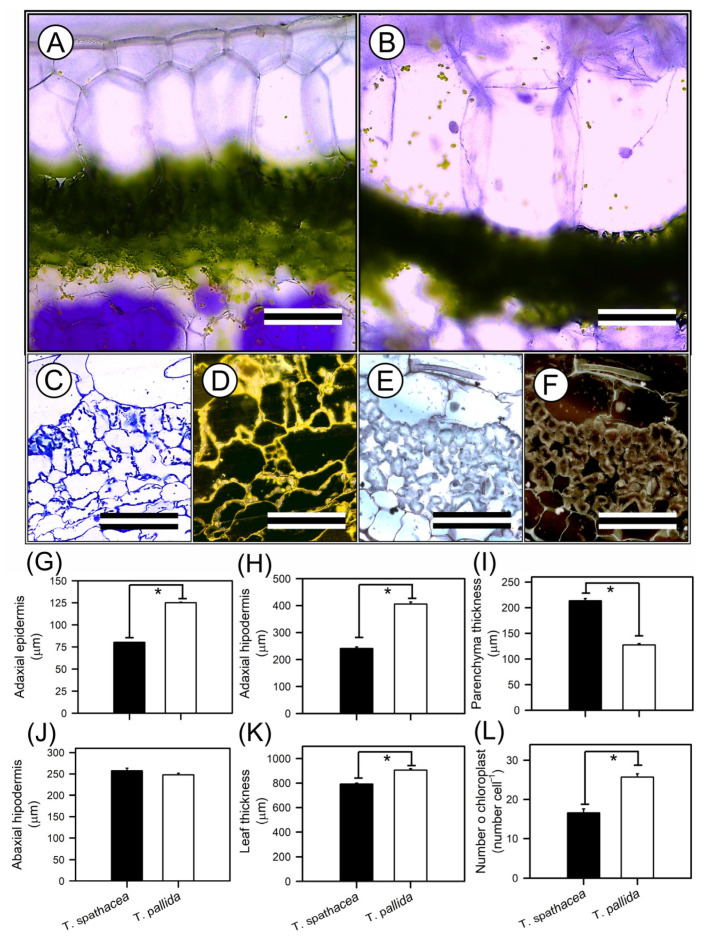
Representative optical microscopy (OM) images of the top–bottom and anatomical analyses of *Tradescantia spathacea* (L.) Olof Swartz and *Tradescantia pallida* (Rose) D.R. Hunt plants. (**A**,**C**,**D**) Cross sections of *T. spathacea* plants. (**B**,**E**,**F**) Cross-sections of *T. pallida* plants. (**G**) Adaxial epidermis. (**H**) Adaxial hypodermis. (**I**) Parenchyma thickness. (**J**) Abaxial hypodermis. (**K**) Leaf thickness. (**L**) Number of chloroplasts. Bars with asterisks show significant differences by Student’s *t* test (*p* < 0.01). Scale bars = 200 μm and 60 μm, from top to bottom. Mean ± SE. (*n* = 100).

The tissues and cells of the leaves presented the anatomical features of *T. spathacea* and *T. pallida*, as visualised by optical microscopy (Figure 4A–L). Cross-sectional imaging revealed structural differences and cellular reorganisation patterns between the species (Figure 4A–F). The adaxial epidermis of *T. spathacea* (Figure 4A,G), measuring 80.25 µm^2^, was significantly thinner (*p* < 0.01) than that of *T. pallida*, measuring 125.08 µm^2^ (Figure 4B,G). Conversely, the adaxial hypodermis of *T. spathacea* (240.87 µm^2^) was less extensive than the 405.85 µm^2^ observed in *T. pallida* plants (Figure 4A,B,H).

The parenchyma thickness in *T. spathacea* (257.38 µm^2^) increased compared with that in *T. pallida* (247.78 µm^2^), revealing a denser mesophyll layer that may enhance photosynthetic efficiency (Figure 4C–F,I). The compact cells of the mesophyll parenchyma in *T. spathacea* indicated limited cellular expansion and a high concentration of chloroplasts, which may restrict light passage in deep layers (Figure 4C–F, and see purple points in hypodermal cells).

The abaxial hypodermis of *T. spathacea* was 213.50 µm^2^ thinner than that of *T. pallida* (127.48 µm^2^) (Figure 4J). Higher concentrations of extrachloroplastic pigments, such as anthocyanins, were observed in these cell layers (Figure 4D,F,J).

The total leaf thickness of *T. spathacea* (792 µm^2^) contrasts with the greater thickness of *T. pallida* (906.18 µm^2^) (Figure 4K), showing differential cell structures to support the accumulation of water and pigments in large vacuoles in plant cells (Figure 4A–F).

Chloroplast density was greater in *T. pallida* (25.69 chloroplasts cells^−1^) than in *T. spathacea* (16.59 chloroplasts cell^−1^) (Figure 4C–F,L), which could be correlated with the species’ photosynthetic activity and adaptation to light conditions, indicating a balance between the number of chloroplasts and the thickness and area of the chloroplastic and nonchloroplastic cellular regions (Figure 4).

### 3.4. Leaf Anatomy Ultrastructures by Transmission Electron Microscopy

**Figure 5:**
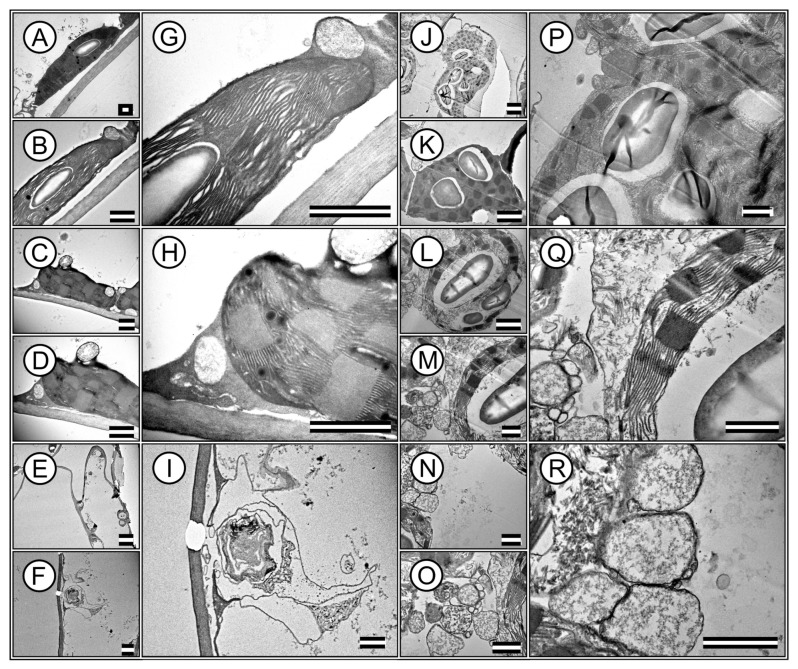
Representative transmission electron microscopy (TEM) image of ultrastructures in mesophilic cells of *Tradescantia spathacea* (L.) Olof Swartz and *Tradescantia pallida* (Rose) D.R. Hunt plants. (**A**–**I**) *T. spathacea* species. (**J**–**R**) *T. pallida* species. Stromal and granal thylakoids are evidenced. Accumulation of cell wall, starch and chloroplasts and vesicles accumulate different substances based on electrodensity. Scale bars = 1 μm.

Transmission electron microscopy (TEM) images revealed ultrastructures within the palisade parenchyma and hypodermal cells. High-resolution TEM enables in-depth analysis of cellular components, providing insight into the ultrastructural adaptations of plant cells (Figure 5A–R).

The TEM images highlight the integrity and distribution of chloroplasts, which are densely packed within the cells. This organization in *T. spathacea* (Figure 5A,B,G) compared with that in *T. pallida* (Figure 5J,K,P) showed a possible efficient photosynthetic apparatus with many thylakoid membranes that are crucial for modifying leaf spectral absorption (Figure 5).

Remarkably, the chloroplasts exhibited a high degree of structural organisation, with clearly defined granum stacks, indicating a substantial capacity for energy capture and transfer and electron-dense membranes (Figure 5G,H,Q). The presence of large and abundant starch grains within the chloroplasts indicated active and higher photosynthesis and carbohydrate synthesis (Figure 5G,P). Various organelles essential for cell function were visible in the cytoplasmic matrix (Figure 5D,H,M,Q). Mitochondria, the sites of cellular respiration and energy production, were identifiable, revealing an integral link between energy-producing and energy-utilising processes within the cell, and no plastoglobules were identified (Figure 5C,D,H,L,M,Q).

Additionally, the images revealed that the cell wall was thick and robust, contributing to cellular rigidity and structural integrity, particularly in *T. spathacea* (Figure 5H). However, the cell wall is robust in *T. pallida*, with major cytoplasmic and minor vacuoles in the cytoplasm.

Nevertheless, other images from the apical meristematic region of *T. spathacea* showed chloroplasts at various stages of degradation, with vestiges of the membranes (Figure 5E,F,I). In contrast, in *T. pallida*, no denatured membranes were observed; instead, a series of vesicles and vacuoles contained electron-dense substances (Figure 5N,O,R).

The hypodermis of *T. spathacea* (Figure 5E,F,I) and *T. pallida* (Figure 5N,O,R) exhibited clustered vacuoles containing deposited material in addition to disorganised membranes. This indicates that any chloroplasts present have become nonfunctional, given the characteristics of the accumulation of solutes and water and the deposition of secondary metabolic materials (Figure 5).

**Figure 6:**
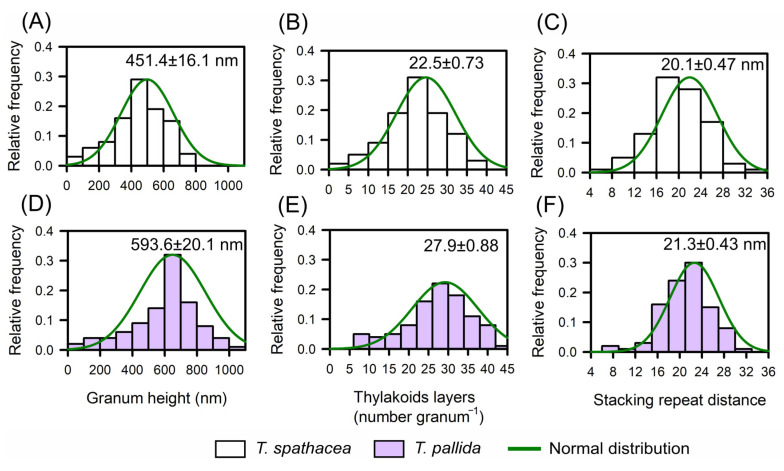
The relative distribution of frequencies according to biophysical parameters aligns the ultrastructures of the chloroplasts and thylakoids of *Tradescantia spathacea* (L.) Olof Swartz and *Tradescantia pallida* (Rose) D.R. Hunt plants. (**A**) Relative distribution of the frequency of *T. spathacea* granum height (nm). (**B**) Relative distribution of the frequency of thylakoid layers in *T. spathacea*. (**C**) Relative distribution of the frequency of stacking repeats in *T. spathacea* species. (**D**) Relative distribution of the frequency of granum height (nm) in *T. pallida* species. (**E**) Relative distribution of the frequency of thylakoid layers in *T. pallida*. (**F**) Relative distribution of the frequency of stacking repeat distances in *T. pallida* species. The green line shows the normalised distribution. (*n* = 100).

Base TEM was used to determine the relative frequency distributions of the biophysical parameters defined in *T. spathacea* and *T. pallida* species based on the chloroplast distribution and thylakoid layers (Figure 6A–F). Histograms juxtaposed with a normal distribution curve provided a statistical view of the granum height, thylakoid layers, and stacking repeat distance (Figure 6).

For *T. spathacea*, the average granum height was 451.4 ± 16.1 nm (Figure 6A), indicating the vertical extent of stacked thylakoid membranes within the chloroplasts. The frequency of thylakoid layers averaged 22.5 ± 0.73 per granum (Figure 6B), which reflects the number of thylakoid membranes per stack, and the stacking repeat distance—the space between successive thylakoids—was 20.1 ± 0.47 nm (Figure 6C).

Comparatively, *T. pallida* has a different structural composition, with a granum height of 593.6 ± 20.1 nm (Figure 6D), demonstrating taller granum stacks than those observed in *T. spathacea* species. The average number of thylakoid layers was 27.9 ± 0.88 per granum (Figure 6E), indicating a greater number of membranes per stack, and the average stacking repeat distance was 21.3 ± 0.43 nm (Figure 6F).

Histograms revealed that compared with *T. spathacea*, *T. pallida* typically has taller granum stacks with more thylakoid layers and a slightly greater stacking repeat distance (Figure 6C,F). These characteristics of thylakoids in chloroplasts have profound implications for the photosynthetic and antioxidant adaptability of plants, influencing their capture, scattering, and use of light energy in leaf cells.

### 3.5. Pigment Components in Cell Structure and Ultrastructure

**Figure 7:**
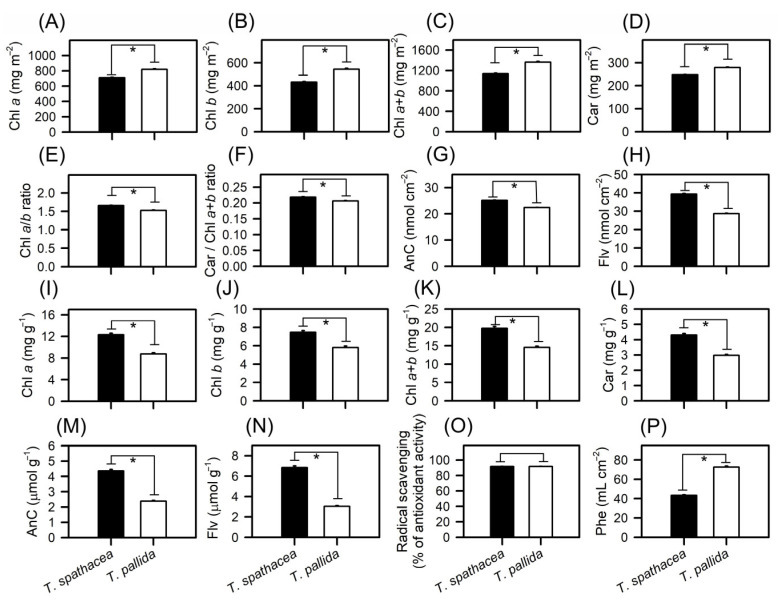
Concentrations of compounds in *Tradescantia spathacea* (L.) Olof Swartz and *Tradescantia pallida* (Rose) D.R. Hunt plants. (**A**) Chlorophyll *a* (mg m^−2^). (**B**) Chlorophyll *b* (mg m^−2^). (**C**) Total chlorophyll (*a*+*b*) (mg m^−2^). (**D**) Carotenoids (mg m^−2^). (**E**) Chl *a*/*b* ratio. (**F**) Car/Chl *a*+*b* ratio. (**G**) Anthocyanins (nmol cm^−2^). (**H**) Flavonoids (nmol cm^−2^). (**I**) Chlorophyll *a* (mg g^−1^). (**J**) Chlorophyll *b* (mg g^−1^). (**K**) Total chlorophyll (*a*+*b*) (mg g^−1^). (**L**) Carotenoids (mg g^−1^). (**M**) Anthocyanins (μmol g^−1^). (**N**) Flavonoids (μmol g^−1^). (**O**) Radical scavenging (g gallic acid^−1^). (**P**) Phenolic compounds (mL cm^−2^). Bars with asterisks show significant differences by Student’s *t* test (*p* < 0.01). Mean ± SE. (*n* = 100).

A comparative analysis of the pigment content in *T. spathacea* and *T. pallida* was conducted by extracting pigments from both chloroplastidic and extrachloroplastidic cells (Figure 7A–P). Analysis revealed that the concentrations of chlorophyll *a* (Chl*a*) and chlorophyll *b* (Chl*b*) in *T. spathacea* were greater than those in *T. pallida*, with values of 709.91 mg m^−2^ and 430.92 mg m^−2^, respectively (Figure 7A,B). This was also reflected in the total chlorophyll (Chl*a*+*b*) content, which was 1140.82 mg m^−2^ in *T. spathacea* (Figure 7C). The carotenoid content in *T. spathacea* was 247.88 mg m^−2^, which was significantly lower than that in *T. pallida* (279.20 mg m^−2^) (Figure 7D) (*p* < 0.01).

The *Chla/Chlb* ratio in *T. spathacea* was greater than the Chl*b*, which is consistent with the high photosynthetic capacity of the chloroplasts and extrachloroplastidic plant cells (Figure 7E). Conversely, *T. pallida* displayed a lower carotenoid-to-chlorophyll ratio (Car/Chl*a*+*b*), indicating a different balance of these pigments, which may reflect the varied light-harvesting strategies (Figure 7F).

Anthocyanin levels showed significant interspecies variation, with *T. spathacea* exhibiting high concentrations both per base area and per mass of leaf, at 25.17 nmol cm^−2^ and 4.36 µmol g^−1^, respectively (Figure 7G,M). Similarly, the flavonoid content was greater in *T. spathacea*, at 39.36 nmol cm^−2^ and 6.84 µmol g^−1^ (Figure 7H,N). On the other hand, *T. pallida* exhibited lower values for these same pigments with Chl*a*, Chl*b*, Chl*a*+*b*, Car, AnC and Flv per mass, even though its colouration is “visually” more purple leaves (Figure 7A–N).

Photochemical efficiency, assessed by radical scavenging activity, revealed that *T. spathacea* has a significantly greater capacity for radical scavenging, indicating the potential for greater antioxidant activity within this species (Figure 7O). The data showed an increase of approximately 70% in the radical scavenging activity compared to that of *T. pallida* species. The phenolic compound concentration measured per base area further supported this finding, with *T. spathacea* exhibiting a concentration approximately 50% higher than that of *T. pallida* (Figure 7P), demonstrating significant differences (*p* < 0.01).

### 3.6. Leaf Optical Profile and Absorbance Spectrum of Pigments

**Figure 8:**
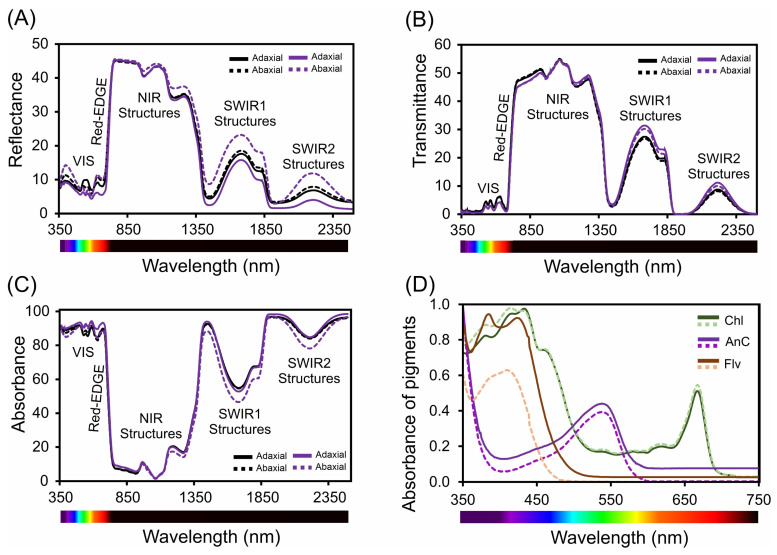
Spectral analysis of leaves (*in vivo*) and pigments (*in vitro*) in *Tradescantia spathacea* (L.) Olof Swartz and *Tradescantia pallida* (Rose) D.R. Hunt plants. (**A**) Reflectance factor (Ref) from 350 to 2500 nm. (**B**) Transmittance factor (trans) from 350 to 2500 nm. (**C**) Absorbance factor (Abs) from 350 to 2500 nm. (**D**) Spectra of chloroplast (chlorophylls and carotenoids) and extrachloroplastidic (anthocyanins and flavonoids) pigments from 350 to 750 nm. The solid and dashed black lines represent the measurements from the adaxial and abaxial surfaces of the leaves of *Tradescantia* species, respectively. (*n* = 100).

The leaf optical profile differed between the adaxial and abaxial surfaces, as did the spectral characteristics of reflectance, transmittance, and absorbance (Figure 8A–D and Figure S1).

In the ultraviolet (UV) spectrum (350–400 nm), we observed low reflectance, which is indicative of effective UV light absorption (Figure 8A and Figure S1A). Specifically, the lowest reflectance values at 380 nm were 5.6% for *Tradescantia spathacea* and 6.3% for *Tradescantia pallida* on the adaxial surfaces. A distinct reduction in the reflectance of *T. spathacea* on the adaxial surface was observed at violet/blue wavelengths (400–450 nm), which was 12% lower than that of its abaxial counterpart. In addition, the interaction of light with biochemical and biophysical compounds results in distinct reflectance patterns associated with cell surface characteristics as well as interactions with the upper cellular layers of the epidermis and hypodermis (Figure 8A, see details in reflectance spectra). The red range (620–700 nm) exhibited a pronounced decrease in reflectance, notably at 680 nm, where *T. spathacea* and *T. pallida* exhibited adaxial reflectance values of 11% and 13%, respectively (Figure 8A and Figure S1A).

The low transmittance in the UV region suggests a unique adaptation within the *Tradescantia* species-based concentration of pigments in the cells (Figure 8A and Figure S1B). As the spectrum transitions to violet/blue, the decrease in transmittance, particularly for *T. spathacea* on the adaxial surface by 10% at 450 nm, aligns with the increased absorption of the photoprotective and antioxidant compounds (Figure 8B, range for spectral bands). The transmittance continues to decrease toward the red end, paralleling the trend in which the reflectance aligns with that of interactions such as denatured chloroplasts and membranes, cell walls, and vacuoles containing compounds of different densities (Figure 8B, see details on the NIR structures in this range). In the NIR region (700–1000 nm), the transmittance increased for both species, suggesting that the structural aspects of the leaves facilitated light passage. In parallel, *T. pallida* showed a significant increase in transmittance of 15% in the SWIR2 band (1800–2500 nm) compared to that of *T. spathacea*, suggesting that differences in leaf water content or density are associated with the presence of many vacuoles in cells (Figure 4B).

The absorbance spectra revealed increased absorption across the UV spectrum for both *Tradescantia* species, with *T. spathacea* on the adaxial surface absorbing up to 90% of UV light (Figure 8A and Figure S1C). This strong UV absorbance is likely a protective adaptation against harmful radiation. In the violet/blue region, the absorbance at 430 nm and decrease in the intensity of the green band (530–560 nm) were particularly high for *T. spathacea*, reflecting the presence of anthocyanins and other blue–green light-absorbing pigments. The absorbance peaks were within the visible region, particularly at a red wavelength of 680 nm (*T. spathacea* adaxial: 92%). The absorbance in the NIR region notably decreased to 15% for *T. spathacea* and 18% for *T. pallida* at 750 nm, which is indicative of decreased pigment interaction and translucency of plant tissues at these wavelengths (Figure 8C), but dispersion of light in the interiors of chloroplasts, cells, and leaves (Figure 8C).

In the SWIR1 (1300–1800 nm) and SWIR2 (1800–2500 nm) regions, the absorbance trends varied less markedly between the two leaf sides, suggesting interactions with leaf components beyond the pigments predominantly responsible for adaptations to drought environment, such as water, which has distinct absorbance characteristics in the SWIR range and can indicate water status and cell structure integrity for cellular homeostasis (Figure 8A–C).

The absorption spectra of the pigments within plant leaves are shown in Figure 8D. The spectral data correspond to the absorbance characteristics of different pigments, such as chlorophyll, anthocyanins, and flavonoids, across the visible-NIR proximal light spectrum (Figure 8D). The chlorophyll curve shows peaks in the blue (approximately ≈ 430–450 nm) and red (approximately ≈ 660–680 nm) regions, which are typical absorption wavelengths for chlorophyll and reflect the role of pigments in capturing light energy for photosynthesis. The intensity of the peak at approximately 530 nm indicates the absorption properties of anthocyanins, which contribute to leaf colouration and photoprotection. The flavonoid absorption curve spanned a broader range, suggesting that absorption in the UV-cyan band extends into the visible region, which is consistent with the roles of flavonoids in UV protection and oxidative stress mitigation (Figure 8D), and the reflectance, transmittance, and absorbance of flavonoids are correlated with the leaf spectrum (Figure 8A–D). The spectral patterns provided insights into the plant’s light harvesting and protective mechanisms, for example, with specific absorption maxima and minima revealing detailed information about the types and concentrations of pigments and their metabolic pathways such as the possible increase of the pathways of flavonoids, anthocyanins, and phenolic compounds (Figure 8A–D).

### 3.7. Chlorophyll a Fluorescence Kinetics-Based Pigments and the Structure and Ultrastructure of Cells

**Figure 9:**
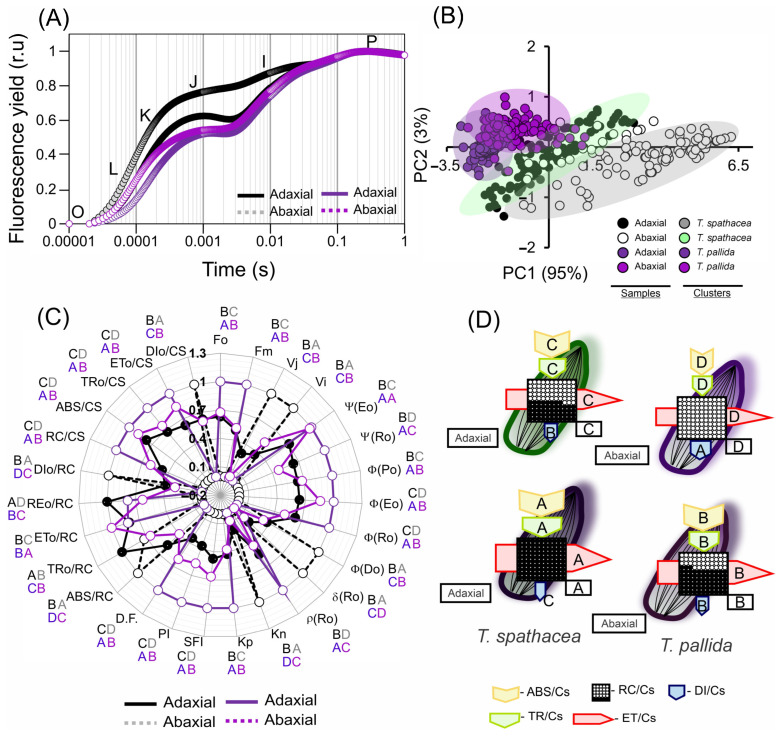
Chlorophyll *a* fluorescence kinetic parameters derived from the JIP test of *Tradescantia spathacea* (L.) Olof Swartz and *Tradescantia pallida* (Rose) D.R. Hunt plants. (**A**) Kinetics of chlorophyll *a* fluorescence induction with normalisation. (**B**) Principal component analysis of the adaxial and abaxial leaf surfaces. (**C**) Radar plot displaying parameters derived from the transient chlorophyll *a* fluorescence kinetics (JIP test). (**D**) Pipeline variegatum leaves displaying phenomenological energy flow through excited leaf cross sections (CSs). Yellow arrow: ABS/CS, absorption flow by approximate CS; green arrow: TR/CS, energy flow trapped by CS; red arrow: ET/CS, electron transport flow by CS; blue arrow: DI/CS, energy flow dissipated by CS; circles inscribed in squares: RC/CS indicate the percentage of active/inactive reaction centres. The white circles inscribed in squares represent reduced (active) QA reaction centres, the black circles represent non-reducing (inactive) QA reaction centres, and 100% of the active reaction centres responded with the highest average numbers observed in relation to the control. The arrow size indicates the change in energy flow compared with that of the green surface leaves in *T. spathacea*. Different letters and colours indicate significant differences between plants, as determined using Duncan’s test (*p* < 0.01). The standard error was omitted for clarity. (*n* = 100).

Analysis of chlorophyll *a* fluorescence kinetics for *T. spathacea* and *T. pallida* based on the JIP test parameters revealed distinct differences between the adaxial and abaxial faces and cells (Figure 9A–D). The fluorescence induction kinetics, observed through the normalised OJIP transient, flash-pulse induction, modulated the double emission of the adaxial and abaxial faces of the species (Figure 9A–D).

The fluorescence curves indicate a greater proportion and differences in the K-J-I bands, stabilising and reaching a plateau in the P-bands (Figure 9A). In addition, the principal component data (PC: 95% and PC2: 3%) explained 98% of the total variability of the data when analysing the adaxial and abaxial faces of the species (Figure 9B).

In the analysis of the JIP test by photosynthetic parameters of the *T. spathacea* and *T. pallida* species, both the adaxial and abaxial surfaces were examined in comparison with the standard *T. spathacea* adaxial (Figure 9C,D; green surfaces). Ψ(Eo) in the *T. pallida* adaxial increased by 16.99%, with the abaxial surface showing an increase of 15.66%, whereas *T. spathacea* abaxial decreased by 40.88%. Ψ(Ro) values increased in *T. pallida* adaxial by 9.33% and decreased in *T. spathacea* abaxial by 16.57%. ϕ(Po), a measure of maximum quantum yield, increased in the *T. pallida* adaxial by 9.79% and marginally in its abaxial by 1.19%, indicating more efficient primary photochemistry than that of *T. spathacea* (Figure 9C).

In terms of the ϕ(Eo) parameter, which pertains to the quantum yield of electron transport, adaxial *T. pallida* demonstrated an increase of 27.79% with abaxial *T. pallida* at 16.91%. This suggests the existence of a more robust electron transport chain. However, for ϕ(Ro), the quantum yield of electron transport to reduce the end electron acceptors at the PSI acceptor side, the *T. pallida* adaxial only showed a slight increase of 17.24%, whereas *T. spathacea* abaxial showed a reduction of 5.37% (Figure 9C).

For ϕ(Do), which indicates the quantum yield of non-photochemical energy dissipation in PSII, there was a significant decrease in *T. pallida* adaxial by 22.62%. δRo, which expresses the efficiency with which an electron from the intersystem electron carrier is transferred to reduce the end electron acceptors at the PSI acceptor side, was reduced in the *T. pallida* adaxial by 4.57%, whereas the *T. spathacea* abaxial decreased by 13.84% (Figure 9C).

The ρRo values, reflecting the probability that an electron from the intersystem electron carrier moves further than PSI, were elevated by 31.30% in *T. pallida* adaxial and decreased by 7.57% in *T. spathacea* abaxial. Both Kn and Kp, denoting the de-epoxidation state of xanthophyll cycle pigments, were slightly reduced in *T. pallida* adaxial by 6.14% and 20.69%, respectively (Figure 9C).

The Structure Function Index (SFI) increased by 59.48% in *T. pallida* adaxial, indicating the enhanced structural integrity of the thylakoid membranes. The Performance Index (PI), encompassing overall photosynthetic performance, was notably higher in the adaxial face of *T. pallida* than in the abaxial face by 117.52% and 36.40%, respectively. Finally, the Dissipation Factor (D.F.) in the *T. pallida* adaxial increased by 68.02% with a more moderate increase of 27.51% in the abaxial face, signifying a higher energy dissipation as heat, potentially indicating stress or a higher capacity for non-photochemical quenching (Figure 9C).

Based on radar plots (Figure 9C) and phenological flux models (Figure 9D), in *T. spathacea,* the proportion of active reaction centres (RC/CS), reflecting the number of active photosystem II (PSII) units per cross-sectional area, was significantly greater, with an average of 47.22%, as opposed to the absence of active reaction centres on the abaxial surface of the same species. This percentage reached 100% on the adaxial surface and 60.41% on the abaxial surface of *T. pallida*, suggesting a greater density of PSII on the abaxial surface of *T. pallida* (Figure 9C).

The absorption per cross-section (ABS/CS) of *T. spathacea* was 9.29% on the adaxial surface and 5.99% on the abaxial surface, whereas that of *T. pallida* was 11.97% and 10.56%, respectively. This parameter measures the light-harvesting efficiency of chlorophyll *a* in photosystems (Figure 9C,D).

The percentage of energy trapped per cross section (TRo/CS) was 7.03% in *T. spathacea* on the adaxial surface and 5.23% on the abaxial surface, with *T. pallida* displaying percentages of 10.45% and 7.91%, respectively (Figure 9C,D).

In addition, the percentages of electron transport per cross-section (ETo/CS) for the adaxial and abaxial surfaces of *T. spathacea* were 4.50% and 3.59%, respectively, whereas those of *T. pallida* were 7.18% and 6.06%, respectively, which reflects the rate at which electrons are transported beyond QA^−^ (Figure 9C,D).

The dissipation energy flow per cross-section (DIo/CS) was lower in *T. spathacea* (3.38% on the adaxial surface and 5.41% on the abaxial surface), indicating lower NPQ; however, this aligns with the presence of hypodermal cells and the concentrations of anthocyanins, phenolic compounds, and flavonoids (Figure 9D). For *T. pallida*, the values were 2.71% and 3.25% for the adaxial and abaxial surfaces, respectively.

Aligning these data, the radar plot with phenomenological models (Figure 9C,D) provides a visual representation of the JIP test parameters, and these patterns of energy conversion efficiency and NPQ elucidate the different photochemical reaction strategies employed by these species, as evidenced by the fluorescence induction kinetics (Figure 9A–D).

### 3.8. Correlation Coefficients between the Hyperspectral Vegetation Indices and Biophysical Parameters of the Leaves

**Figure 10:**
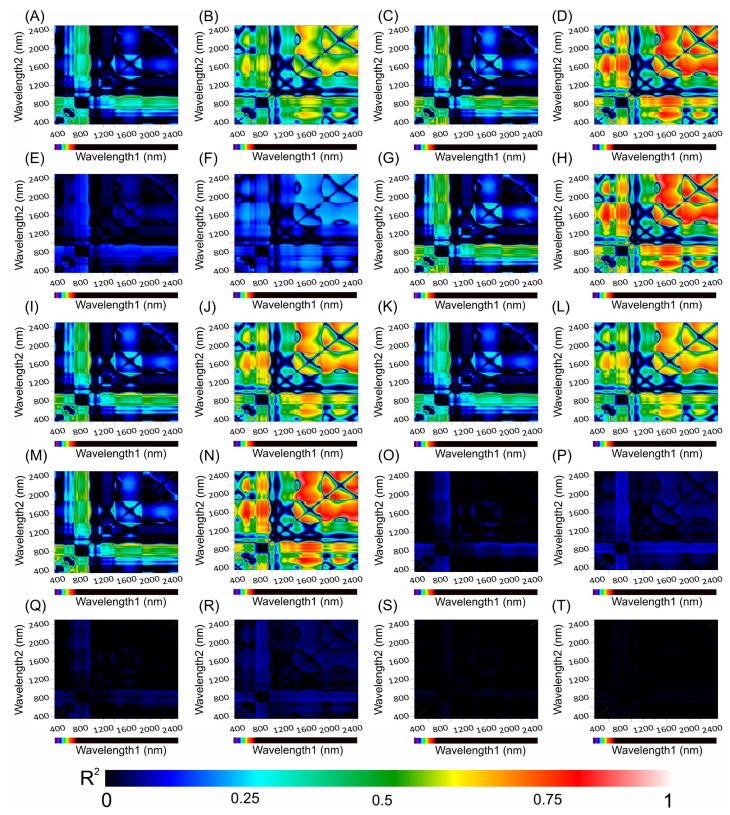
Counter map for correlation coefficients (R^2^) from HVI algorithms applied to adaxial and abaxial absorbance hyperspectroscopy data across the range of 350 to 2500 nm for *Tradescantia spathacea* (L.) Olof Swartz and *Tradescantia pallida* (Rose) D.R. Hunt plants. The heatmap shows the coefficient of correlation (R^2^) obtained from linear regression analyses between various biophysical parameters and the interactions between wavelengths1 and wavelengths2. (**A**,**B**) adaxial epidermis, (**C**,**D**) adaxial hypodermis, (**E**,**F**) parenchyma thickness, (**G**,**H**) abaxial hypodermis, (**I**,**J**) leaf thickness, (**K**,**L**) number of chloroplasts, (**M**,**N**) adaxial cell area surface, (**O**,**P**) granum height, (**Q**,**R**) thylakoid layers, and (**S**,**T**) stacking repeat distance (SRD). The colour gradient, which transitions from dark blue to light red, signifies increasing correlation strength. (*n* = 200).

**Figure 11:**
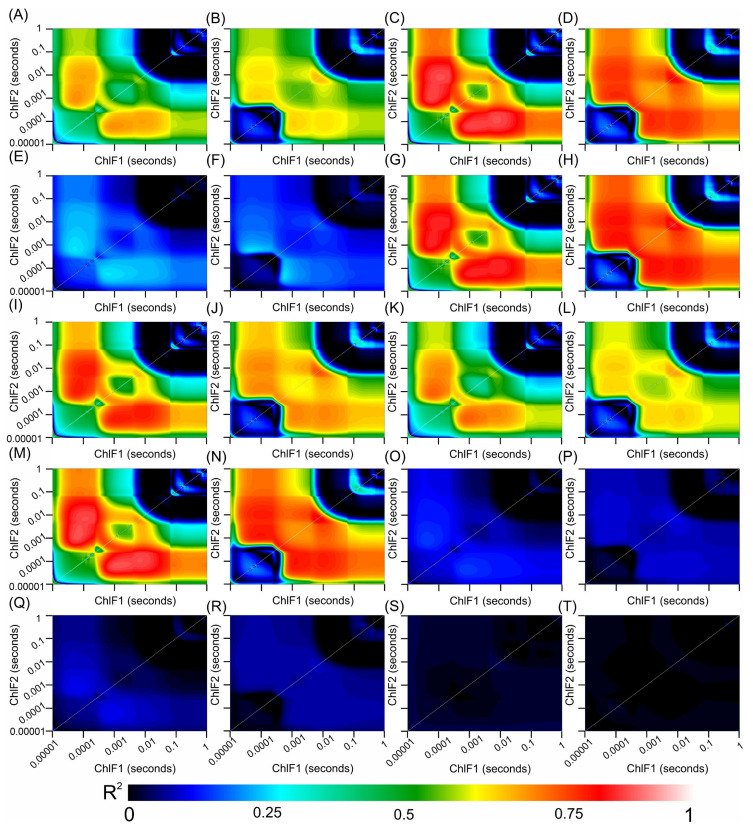
Counter map for correlation coefficients (R^2^) from HVI algorithms applied to adaxial and abaxial chlorophyll *a* fluorescence kinetics data across the range of 350 to 2500 nm for *Tradescantia spathacea* (L.) Olof Swartz and *Tradescantia pallida* (Rose) D.R. Hunt plants. The heatmap shows the coefficient of correlation (R^2^) obtained from linear regression analyses between various biophysical parameters and interactions between wavelengths 1 and 2. (**A**,**B**) adaxial epidermis, (**C**,**D**) adaxial hypodermis, (**E**,**F**) parenchyma thickness, (**G**,**H**) abaxial hypodermis, (**I**,**J**) leaf thickness, (**K**,**L**) number of chloroplasts, (**M**,**N**) adaxial cell area surface, (**O**,**P**) granum height, (**Q**,**R**) thylakoid layers, and (**S**,**T**) stacking repeat distance (SRD). The colour gradient, which transitions from dark blue to light red, signifies increasing correlation strength. (*n* = 200).

Figure 10 and Figure 11 present heatmaps of correlation coefficients from linear regression analyses of hyperspectral vegetation indices (HVIs) spanning a wavelength spectrum from 350 to 2500 nm, including chlorophyll *a* kinetics for *Tradescantia spathacea* and *Tradescantia pallida*, as shown in Figure 10 and Figure 11 (see panels 10 and 11 from A, C, E, G, I, K, M, O, Q, and S for *T. spathacea* and B, D, F, H, J, L, N, P, R, and T for *T. pallida*). The depicted colour gradients illustrate a range of R^2^ values, where the intensity reflects the correlation strength. In addition, the results indicate strong associations between the differential absorption patterns upon illumination and structural correlations within the UV, VIS, NIR, and SWIR bands (Figure 10).

The strongest correlations occurred within the SWIR1 and SWIR2 bands with notable interactions between the VIS and SWIR bands (Figure 10 and Figure 11). However, correlations approaching zero were particularly noticeable for biophysical parameters related to organelle structures or thylakoid membranes, such as granum height (Figure 10O,P), thylakoid layers (Figure 10Q,R), and stacking repeat distance (SRD) (Figure 10S,T), for both species under investigation (Figure 10).

The chlorophyll kinetic correlation maps indicated that regions corresponding to curves K, J, and I were most significantly affected by interactions with cells containing extrachloroplastidic pigments (e.g., anthocyanins and phenolics) rather than those related to the chlorophyll concentration within the chlorophyll parenchyma. Furthermore, the JIP test curves did not show a correlation with the HVIs for thylakoid stacking, granum thickening, or stacking repeat distance among the thylakoid membranes within cellular chloroplasts (Figure 11).

### 3.9. Multivariate Analyses

**Figure 12:**
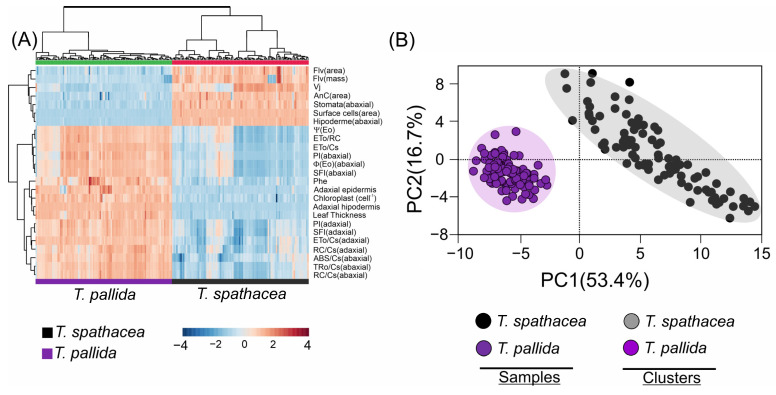
Cluster heatmap and principal component analysis of *Tradescantia spathacea* (L.) Olof Swartz and *Tradescantia pallida* (Rose) D.R. Hunt plants. (**A**) Cluster heatmap summarising pigments by area and mass, chlorophyll *a* fluorescence parameter, and adaxial, abaxial, and anatomical parameters. Numerical differences within the data matrix are shown by the Z scores, where blue and red indicate decreased and increased values, respectively. The parameters were clustered in the rows, and sample groups were clustered in the columns by differences between species at independent factors. (**B**) Principal component analysis and clustering based cluster heatmap factors. (*n* = 100).

A cluster heatmap (Figure 12A) revealed distinct biophysical and biochemical characteristics comparing the *T. spathacea* and *T. pallida* species (Figure 12A). In the heatmap, the colour intensity indicates the level of parameter expression, blue represents lower values, and red indicates higher values. The heatmap uses a Z-score spectrum to demonstrate relative changes in values, organising the data by grouping parameters in rows and sample groups in columns, which accentuates species-specific differences. The variables related to anthocyanins, flavonoid compounds, surface cells, and the abaxial epidermis were strongly correlated with *T. spathacea* and weakly correlated with *T. pallida* (Figure 12A, see red details).

In contrast, the PCA scatter plot summarised the data into two principal components, separating 70.1% of the total explication for the two species (Figure 12B). The closer clustering of *T. spathacea* suggested less variability in the measured parameters, or alternatively, for *T. pallida*, depending on cluster identification. The principal components PC1 and PC2 explained 53.4% and 16.7% of the variance, respectively, reducing complex data into principal factors that describe species-specific traits. PCA distinguished the species and validated their unique biological and structural features (Figure 12B).

## 4. Discussion

### 4.1. Comparative Analysis of Morphological and Biochemical Characteristics

The phenotypic traits of plants are altered through the modulation of gene expression [46,47]. Phenotypic variation in epidermal cells, particularly concerning their interaction with observational patterns of leaf structures and the leaf optical profile, has received little attention [48,49]. Further research is imperative to understand the association between these variations and the cellular spectral signatures at the ultrastructural level [32,50].

In this study, we investigated the morphological and biochemical characteristics of *Tradescantia spathacea* and *Tradescantia pallida* (Figure 1, Figure 2, Figure 3, Figure 4, Figure 5, Figure 6, Figure 7, Figure 8, Figure 9, Figure 10, Figure 11 and Figure 12). We focused on purple and green leaves to demonstrate how the accumulation of pigments (Figure 2), notably anthocyanins and flavonoids, can modify the spectral signatures and chlorophyll patterns on both adaxial and abaxial leaf surfaces [51,52]. Significant differences were observed in the optical properties and fluorescence kinetic parameters of the two species, suggesting that their distinct photosynthetic behaviours were influenced by their morphological traits [53,54]. Notably, the interplay between epidermal cells and leaf pigments revealed unique traits in similar leaves that were discernible upon integrating the spectra at wavelengths of 350–2500 nm [55,56,57,58]. The ultrastructural and cellular components of these species shown above determine their cellular reprograming and subsequent functional pathways by metabolic profile [59,60,61].

Some researchers have shown that spectral and hyperspectral signatures, along with chlorophyll fluorescence, align with morphological alterations [52,62,63,64,65]. This concordance extends to the spectral data and ChlF disparities for various species, albeit through a novel approach. The spectral signatures principally mirror pigment composition (VIS-NIR) and structural adjustments at both the cellular and ultrastructural levels. Chlorophyll fluorescence primarily indicates the photosynthetic efficiency of leaf surface cells, thereby emphasising the functional implications of morphological variation [2,28,30,66,67]. By measuring the fluorescence emitted by a flash or detection between two sensors that penetrated the mesophyll tissue profile, we ascertained that the internal leaf signals diminished upon the sensor return [1,11,14,68]. For example, this determines how light dispersion varies among different cellular layers and the impact of adaxial and abaxial hypodermal cells, which contain purple pigments, on the epidermis. The correlation with the HVI models indicated a pronounced distinction in the signal between the superficial and deeper cellular layers. The fluorescence yield phases K, L, J, and lower in O, I, and P, measured on both leaf sides, revealed that the superficial tissues, despite their greater cross-sectional volume, exhibited markedly reduced photosynthetic electron transport activity compared to deeper tissues such as chlorophyll-rich parenchyma. This discrepancy in the reflected and scattered signals can be attributed to the significant structural differences between the adaxial and abaxial aspects of the *Tradescantia* leaves [8,11].

Microscopic observations revealed that internal leaf structures are intricately designed not only to react to environmental stimuli but also to support the plant’s adaptive physiological strategies (Figure 3, Figure 4 and Figure 5).

### 4.2. Adaptations to Cell and Tissue Structures from Spectral Data

The adaxial and abaxial tissue indicate that the hexagonal and rectangular formats, combined with varying cellular densities in the epidermal layers of both *Tradescantia* species, signify an architectural evolution [8,11]. This development is designed to optimise light absorption and curtail water loss, both of which are essential for survival under diverse lighting conditions [1,29,69,70]. The denser cellular matrix of *T. spathacea* might be an evolutionary response to high-light environments, promoting more efficient light capture and utility because of its compact cellular structure. This dense array could be particularly beneficial for obstructing excessive light penetration, which can be deleterious under strong irradiance [6,51,71].

The thickness of the hypodermis in these species underscores the divergent approaches for retaining water and structural integrity [11]. The thinner hypodermis of *Tradescantia spathacea* suggests a reduced structural investment, potentially to increase adaptability to environmental shifts, while the thicker hypodermis of *Tradescantia pallida* might offer greater mechanical support and moisture retention, indicative of an adaptation to more arid conditions [72,73,74]. Investigations into chloroplast density and spatial orientation have revealed the distinct photosynthetic strategies employed by each species. The increased chloroplast density of *T. pallida* could counterbalance its low chlorophyll content, suggesting a delicate equilibrium (homeostasis) to maximise the light absorption per cellular unit [75,76]. This assumption is bolstered by granum and thylakoid structures, which have evolved in tune with the specific lighting milieu of each species, boosting the energy conversion efficiency.

Chloroplast placement within the palisade parenchyma cells and organelle count per cell correlate with the photosynthetic capacity of each species at the subcellular level [20]. *T. pallida* may exhibit superior photosynthetic performance per cell owing to its elevated chloroplast density, potentially offsetting its reduced overall chlorophyll volume relative to that of *T. spathacea* species. Chloroplast positioning, along with granum stacks and a thylakoid architecture, manifests specific adaptations that augment light capture and energy transduction in energetic compounds (NADPH and ATP, see Figure 9C to JIP test parameters for details) [56,77,78].

Furthermore, the merger of anthocyanins, flavonoids, and additional phenolic substances in the epidermal and subepidermal layers suggests sophisticated photoprotection and antioxidant defence modalities [79,80,81]. These compounds can function as both ultraviolet radiation barriers and reactive oxygen species neutralisers under stress [79,82,83]. The integration of structural data with fluorescence kinetics clarified how *Tradescantia* species customise their energy conversion pathways by aligning them with their structural adaptations.

The differences in fluorescence induction kinetics and JIP test parameters across leaf surfaces highlight the influence of cellular and subcellular frameworks on the quantum yield of electron transport and potentially indicating stress or a higher capacity for non-photochemical quenching [2,68,84,85]. Such insights are vital for elucidating how structural attributes steer physiological processes, including photosynthesis and stress responses. The pronounced J and I phases of the ChlF curves on the adaxial side, with heightened anthocyanin, antioxidant, and phenolic contents, contrasted with the lower levels and diminished vacuole presence in the leaves, particularly under subdued pulsed light [86]. This result is consistent with HVI data and heatmap observations (Figure 10, Figure 11 and Figure 12), suggesting that structural changes in the epidermal and hypodermal cells of leaves could have a dual function: to act as light-focusing lenses, and to regulate light scattering out of the cell if not absorbed by regions with higher cellular chlorophyll levels detected by ChlF and spectroscopic curves (Figure 10 and Figure 11). For example, the light is drawn to a central point within this layer of leaves before it diverges and hits a layer of mesophyll beneath. As a result, the chloroplasts are not exposed to the intensified light at the focal point and the arrangements must be modulated in the granal and stromal thylakoids. The most striking instances of epidermal focusing can be seen in the leaves of tropical understory species. The leaves of plants like *Anthurium* and *Begonia* genus have a velvety look, an optical effect caused by the conical form of the epidermal cells. This conical shape also endows these cells with lens-like properties. While recent focus has been on these and other tropical species, epidermal focusing is also observed in temperate species. These measurements were irrespective of the adaxial or abaxial side and were reciprocally detected by sensors on both leaf surfaces (Figure 1).

The JIP-test, which measures the function of PSII, can provide various parameters, such as Ψ(EO), Ψ(RO), ϕ(PO), ϕ(EO), ϕ(RO), ϕ(DO), δRo, ρRo, Kn, Kp, SFI(abs), PI(abs), and D.F., that reflect different aspects of plant photosynthesis and health in *T. spathacea* and *T. pallida*, both adaxial and abaxial surfaces [19,52,62,63,64,65]. These parameters provide valuable information about photosynthetic performance (Figure 9C). For example, Ψ(EO) and ϕ(PO) indicate changes in photosynthetic efficiency, while δRo and SFI(abs) show how energy is dissipated within the photosystem and photoprotective in leaves [56,77,78].

Such contributions may reveal discrete adaptations in the response to biochemical and biophysical compositional changes. For instance, *Tradescantia spathacea* and *Tradescantia pallida* display significant differences in pigment concentration, spanning chlorophylls, carotenoids, anthocyanins, and flavonoids (Figure 1, Figure 2, Figure 3, Figure 4, Figure 5, Figure 6, Figure 7, Figure 8, Figure 9, Figure 10, Figure 11 and Figure 12). Although pigment concentrations disparities between these species are not as pronounced as those in other genera, such as *Euphorbia*, *Croton*, *Phyllanthus*, *Acalypha*, where pigment concentrations in green or purple leaves differ twentyfold, our results still confirm considerable variation [28,87]. Both species manifested similar scattering traits in the mid-infrared region but exhibited greater variations in the SWIR domain, concordant with other plants where leaf structural features influence light scattering, notwithstanding structural and ultrastructural alterations in plant cells.

### 4.3. Photosynthetic Strategies and Cellular Adaptations

The distribution and concentration of important biochemical constituents, such as chlorophylls, carotenoids, lignin, ferulic acid, and proteins, varies within the leaf architecture of *Tradescantia spathacea* and *Tradescantia pallida* (Figure 1, Figure 2, Figure 3, Figure 4, Figure 5, Figure 6, Figure 7, Figure 8, Figure 9, Figure 10, Figure 11 and Figure 12). Differential pigment accumulation not only augments the visual appearance of these species but also significantly contributes to primary and secondary metabolic processes within the leaves [12,88]. The kinetic fluorescence emission properties of chlorophyll *a* in Tradescantia leaves were similar to those of typical plant cells in high absorption areas [2,8,9,10,11,14]. However, regions with intensified pigment concentrations, such as those in purple leaves, exhibited altered absorbance and fluorescence emission owing to self-absorption across all measurement configurations [8,9,14].

The morphology of the adaxial and abaxial epidermal cells, as well as the respective hypodermal layers, profoundly influenced the light absorption and scattering qualities of the leaves [8,9,10]. Because of their distinct optical attributes, these cells function as pivotal barriers and regulators of light ingress. Such an architectural design is crucial in dictating light distribution across the leaf surface, consequently affecting photosynthetic efficiency and photoprotection mechanisms [89,90]. In both *Tradescantia* species, parenchymal cells encompassed within specific cellular frameworks were instrumental in defining plant photosynthetic capacities and xanthophyll cycle pigments and capacity for non-photochemical quenching changes.

Chloroplasts, as organelles housing essential pigments such as chlorophylls and carotenoids, are integral to light absorption for photosynthesis, whereas vacuoles contain anthocyanins and flavonoids for directional light scattering and photoprotection [17,34,68,91]. Varied chlorophyll *a*/*b* ratios and carotenoid quantities across species indicate differing approaches to light energy utilisation, possibly as an adaptive response to their distinct lighting environments [2,92,93]. Furthermore, the elevated anthocyanin and flavonoid levels in *Tradescantia spathacea* suggest a robust ability to mitigate UV damage and oxidative stress. These compounds, which are present in epidermal and hypodermal cells, filter out detrimental radiation while safeguarding the photosynthetic apparatus in the deeper tissue layers [68]. The pronounced presence of phenolic compounds in *Tradescantia spathacea* also signifies a formidable defence mechanism, affording antioxidant advantages that bolster the plant’s resistance to biotic and abiotic stressors [79,93,94,95]. This adaptive trait may enhance the resilience and ornamental value of *T. spathacea*, thereby increasing its versatility for cultivation in diverse environmental settings. Photochemical efficiency, gauged by radical scavenging activity, demonstrated the heightened radical scavenging capacity of *T. spathacea*, implying the potential for superior antioxidant activity within this species (Figure 5O). This was corroborated by the higher phenolic compound concentration and radical scavenging per base area in *T. spathacea*, which significantly surpassed that in *T. pallida* (Figure 5P), indicating distinct interspecies differences (*p* < 0.01). Overall, *Tradescantia pallida* harboured lower concentrations of these bioactive compounds despite its darker hue [8,9,10,11]. This finding suggests that assessing visual intensity does not necessarily reflect the actual levels of bioactive compounds and their antioxidant activities [2,92,93]. It is possible that other factors, not evaluated in this study, might significantly alter the spectral signatures. This highlights the need for further research, not only to determine the pigment composition visible but also to explore the contribution and arrangement of these pigments in the leaf optical profile [2,55,56,57,58]. Such research should consider their relationship with structures, locations, and arrangements, thereby emphasizing the importance of the SWIR (Short-Wave Infrared) bands in determining the spectral fingerprints of leaves.

### 4.4. Interaction Implications of Adaxial and Abaxial Cell Surfaces in Leaves

The understanding of pigment-based adaptations in ornamental plants sets the stage for further research on how these adaptation pathways respond to ultrastructural challenges [8,9,10,11,14]. For example, analysis of pigment distribution on the upper and lower leaf surfaces, along with the role of cellular components in pigment localisation, provides crucial insights into the intricate interplay between plant structure and biochemical pathways for mesophyll cells for adaptation [96].

In summary, the unique pigment profiles and associated biochemical and biophysical properties observed in *Tradescantia spathacea* and *Tradescantia pallida* contribute significantly to our knowledge of plant adaptation mechanisms [79,93,94,95]. This refined understanding has important ramifications for horticulture, particularly for the cultivation and breeding of ornamental plants, and opens new avenues for exploring plant stress physiology and ecological resilience in other genera and families of plants.

Environmental stressors such as high light intensity, salinity, and drought are known to reduce the activity of PSII electron transport *in vivo* [29,97,98]. Our findings suggest that leaf structural changes under environmental stress, such as cellular miniaturisation and less prominent columnar cells, might enhance the refraction of incident light, thereby affecting the J and I phases of the chlorophyll fluorescence induction (ChlF) curves and potentially reducing photosynthetic electron transport activity, as suggested by the JIP test parameters. Alterations in PSII activity can be gauged more accurately by accounting for the potential influence of leaf structure on light refraction. Thus, earlier research may not have fully captured the extent of PSII electron transport activity changes under various stress conditions, based on the presence of vacuoles in cells.

Differences or variations in leaf structure could influence the configuration of ChlF curves in *Tradescantia* leaves, which in turn might affect the assessment of PSII electron transport activity. This hypothesis is supported by the absorption spectra and HVI correlation patterns shown in Figure 8, which revealed significant differences in the reflectance, transmittance, and absorbance between the adaxial and abaxial leaf surfaces. Moreover, spectral absorption profiles (Figure 10) and chlorophyll fluorescence mapping (Figure 11) corroborated this finding, illustrating that the physiological and structural nuances of leaves are strongly related to functional photosynthetic parameters. These findings were further reflected in the heatmap data correlations (Figure 12), providing a comprehensive understanding that transcends chlorophyll activity [19,79,93,94,95].

Further work is required to understand the interaction process between cells and the leaf profile and fluorescence based on variations, such as in albino leaves or those that accumulate anthocyanins solely in plant cells, and how this interaction affects cellular biology and, therefore, the stress responses of developing organs when fingerprints are evidenced in spectral and fluorescence curves. The data presented here suggest that the answer lies, at least in part, in changes in the metabolism of phenolic compounds, such as anthocyanins, flavonoids, antioxidant capacity, and phenolic compounds, but also in structural modifications of the leaf, such as the cellular wall and organelles that are accelerated by processing leading to genetic, molecular and metabolic reprogramming aligned with changes in the leaf’s optical profile.

## 5. Conclusions

This study explored the integration of hyperspectral and chlorophyll *a* fluorescence kinetics sensors to analyse biophysical and biochemical patterns, focusing on the structural and ultrastructural differences in the optical leaf patterns of the two *Tradescantia* species. The data demonstrated a clear correlation between the hyperspectral readings and chlorophyll fluorescence (ChlF) parameters using hyperspectral vegetation indices (HVIs) that aligned the biophysical parameters, revealing a hypoderm with modified fingerprints in the spectral curves. The developed models were highly precise and underscore the efficacy of the multivariate statistical methods employed. Specifically, spectral regions ranging from ultraviolet and blue in the VIS bands to shortwave infrared and SWIR bands proved essential for non-invasive assessments of plant physiology, particularly those associated with significant modifications in the hypodermal cells influencing the hyperspectral fluorescence curves of these plants.

The use of HVIs is crucial for selecting appropriate wavelengths, which leads to enhanced predictive outcomes in estimating cellular changes, including cell surface changes in adaxial and abaxial leaves. The accuracy achieved by these measure underscores the promising role of hyperspectral sensors in advancing our understanding of plant photosynthesis and the distribution of purple pigments in the leaves.

For future research, it would be beneficial to expand the study to include a wider variety of plant species and to incorporate additional analytical methods. This approach enhanced the accuracy and applicability of hyperspectral sensors in plant biology. Moreover, evaluating this approach in diverse field settings may provide further insight into its consistency and versatility, particularly for other plants that accumulate secondary metabolic compounds and possess variegated leaves.

## Data Availability

Data are contained within the article and Supplementary Materials.

## Supplementary Materials

The following supporting information can be downloaded at: https://www.mdpi.com/article/10.3390/cells13110952/s1, Figure S1. Spectral analysis of leaves (*in vivo*) in *Tradescantia spathacea* (L.) Olof Swartz and *Tradescantia pallida* (Rose) D.R. Hunt plants. (**A**) Reflectance factor (Ref) from 350 to 800 nm. (**B**) Transmittance factor (trans) from 350 to 800 nm. (**C**) Absorbance factor (Abs) from 350 to 800 nm. The solid and dashed black lines represent the measurements from the adaxial and abaxial surfaces of the leaves of Tradescantia species, respectively. (*n* = 100).
