# Supplementary material for: Hyperspectral and Chlorophyll Fluorescence Analyses of Comparative Leaf Surfaces Reveal Cellular Influences on Leaf Optical Properties in Tradescantia Plants
Source: Cells. 2024 May 30;13(11):952. doi: 10.3390/cells13110952 (PMC11171972; doi:10.3390/cells13110952)
Supplement: Supplementary file 1 [file cells-13-00952-s001.zip › cells-3001559-supplementary.pdf]

cells

Supplementary Materials

Hyperspectral and Chlorophyll Fluorescence Analyses of Comparative Leaf Surfaces Reveal Cellular Influences on Leaf Optical Properties in Tradescantia Plants

Renan Falcioni [1,*], Werner Camargos Antunes [1], Roney Berti de Oliveira [1], Marcelo Luiz Chicati [1], José Alexandre M. Demattê [2] and Marcos Rafael Nanni [1]

1 Department of Agronomy, State University of Maringá, Av. Colombo, 5790, Maringá 87020-900, Paraná, Brazil; wcantunes@uem.br (W.C.A.); rboliveira@uem.br (R.B.d.O.); mlchicati@uem.br (M.L.C.); mrnanni@uem.br (M.R.N.)
2 Department of Soil Science, Luiz de Queiroz College of Agriculture, University of São Paulo, Av. Pádua Dias, 11, Piracicaba 13418-260, São Paulo, Brazil; jamdemat@usp.br
* Correspondence: renanfalcioni@gmail.com or rfalcioni2@uem.br; Tel.: +55-4430111359

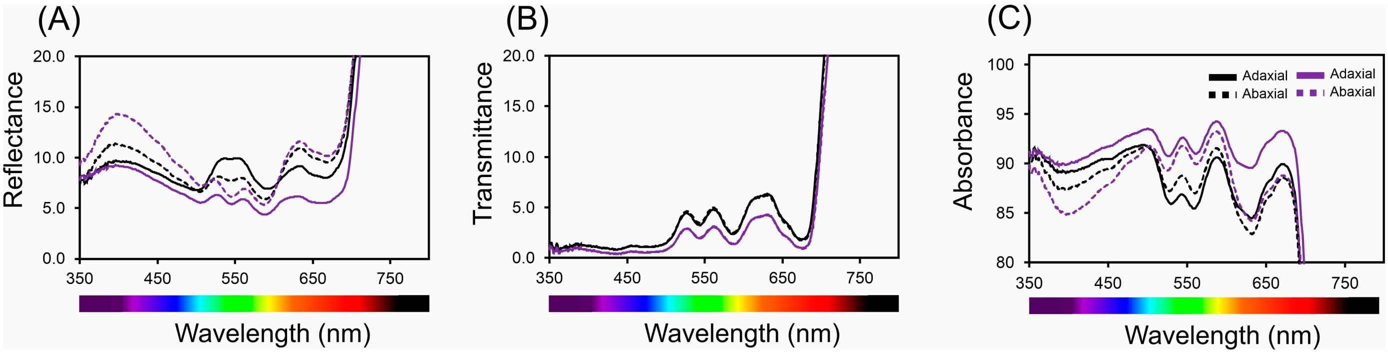

Figure S1. Spectral analysis of leaves (*in vivo*) in *Tradescantia spathacea* (L.) Olof Swartz and *Tradescantia pallida* (Rose) D.R. Hunt., plants (**A**) Reflectance factor (Ref) from 350 to 800 nm. (**B**) Transmittance factor (trans) from 350 to 800 nm. (**C**) Absorbance factor (Abs) from 350 to 800 nm. The solid and dashed black lines represent the measurements from the adaxial and abaxial surfaces of the leaves of *Tradescantia* species, respectively. (*n*=100).
